# Courage, camaraderie and compassion: a qualitative exploration into UK military veterans’ experiences of self-compassion within the context of alcohol use disorders and recovery

**DOI:** 10.1136/military-2023-002383

**Published:** 2023-07-25

**Authors:** Lisa Jane Barrington, A R Bland, J Keenan

**Affiliations:** 1 Medicine Specialty (Alcohol), Manchester University NHS Foundation Trust, Manchester, UK; 2 Psychology, Manchester Metropolitan University, Manchester, UK

**Keywords:** Substance misuse, Suicide & self-harm, PUBLIC HEALTH, COMPLEMENTARY MEDICINE, MENTAL HEALTH, PREVENTIVE MEDICINE

## Abstract

**Introduction:**

UK veterans are at increased risk of mental health and alcohol use disorders (AUDs), experiencing specific challenges such as combat exposure and re-integration which may contribute to treatment barriers. Experiences of shame and AUDs, which may precede or become exacerbated during military service, may be mitigated by self-compassion (SC). This study sought to understand how UK veterans make sense of their SC experiences within the context of their relationships with alcohol and recovery.

**Methods:**

Interpretative phenomenological analysis was used to interpret the SC experiences of five ex-military veterans (one female). Semistructured face-to-face interviews were audio-recorded and transcribed verbatim, with a double hermeneutic approach used to interpret meaningful issues which influenced participants’ self-perceptions in relation to their alcohol use and wider social world.

**Results:**

Two key themes were identified. ‘Searching for Safety’, which illustrated veterans’ SC sense-making within the context of their evolving lifeworld and alcohol use, and ‘Healing with Honour’ which reflected the significance of purpose and identity within experiences of recovery and SC. Findings were interpreted through the lens of the six bipolar elements of SC, which identified SC as salient within veterans’ experiences of AUD and recovery. Although experiences of SC were sometimes perceived as challenging or incongruent to military identity, this was influenced by positive reframing and meaning-making, supported by compassionate narratives and informed trusted relationships.

**Conclusions:**

Veterans’ AUD recovery and support-seeking may be impacted by the experience of SC and enhanced by the early implementation of acceptable and feasible interventions which draw on veterans’ unique military identities and experience. This may include compassion-focussed interventions which reframe SC as fierce SC, peer support models and educational strategies which support healthcare professionals to understand and identify veterans’ military experiences.

**WHAT IS ALREADY KNOWN ON THIS TOPIC**

- UK veterans’ risk of mental health and alcohol use disorders may be exacerbated by the experience of shame, for which self-compassion (SC) is an important and feasible construct to target in this population.

**WHAT THIS STUDY ADDS**

- Veterans’ experience of SC may be developed through interventions, such as mindful reframing, role modelling, peer support and congruent frameworks.
- Fierce self-compassion may be conducive with military identities, while supporting veterans to realise their unique strength and value outside the collective military identity.

**HOW THIS STUDY MIGHT AFFECT RESEARCH, PRACTICE OR POLICY**

- Veterans’ alcohol-related recovery and support-seeking may be impacted by early implementation of compassion-focussed interventions, conducive with military identities.
- Clinicians and policy makers may benefit from understanding veterans’ unique needs and experiences to address the disparity with civilians and to improve integration and transition.

## Introduction

 Research indicates that military veterans experience unique challenges, including re-integration to civilian life,^1^ which may increase their risk of post-traumatic stress disorder (PTSD) and alcohol use disorder (AUD), particularly for those deployed in combat.^2^ Veterans also report a higher prevalence of adverse childhood experiences (ACEs),^3^ which may impact the relationship between military-related challenges, including combat exposure, and current health.^4^ Military enlistment may represent an escape from such adversities^3^ and explain why, despite challenges, veterans often describe the military as a family.^1^ Contrasting the clear expectations and opportunities to excel,^1^ civilian life may lack clarity and purpose,^5^ and elicit contradictory emotions and behaviours from the desire to form comparably strong bonds^6^ with those who may not understand their experiences.^1^

### Alcohol

As military values of discipline, duty and honour become entrenched with self-concept,^7^ support-seeking may appear incongruent with military identity.^1^ In this context, alcohol provides an accessible, efficient anxiolytic for managing emotional, social and vocational problems, and to facilitate camaraderie.^1^ At higher levels, AUD, characterised by increased tolerance, withdrawal symptoms, reduced control and significant impairment,^8^ is associated with emotional avoidance and self-harm.^9^ Often preceding enlistment,^1^ alcohol use may provide an escape from ACEs which often remain unaddressed during military service with limited opportunity to learn effective coping skills.^10^ Highlighting the association between AUD, ACEs and military-related challenges,^4^ veterans often reach crisis point before accessing meaningful support,^7^ reflecting socialised masculine norms of self-reliance and emotional control.

### Shame

In contrast to guilt, which may trigger remorse and reparation,^11^ shame is often internalised, reinforcing beliefs of inferiority and worthlessness.^12^ Often associated with an exaggerated attribution of responsibility,^13^ particularly if reparation cannot be made,^11^ shame may follow combat-related experiences^9^ and/or morally transgressive events; enacting, observing or failing to prevent events which transgress profound moral values.^14^ Shame may increase vulnerability to addictive behaviours through distress avoidance and intensifying feelings of isolation and inadequacy.^15^

### Self-compassion

Self-compassion (SC) is an adaptive affect regulation strategy and an important construct to target in addressing shame-related affect and experiential avoidance^16^ among veterans with AUD-PTSD.^15^ Derived from Buddhist philosophy, SC involves treating oneself with empathy when confronted with perceived shortcomings,^17^ informing compassionate recovery narratives which reframe perceptions from ‘being bad’ to ‘doing bad things’.^15^ Encompassing interrelated facets of self-kindness, common humanity and mindfulness,^18^ SC is pertinent in the contexts of morally transgressive events which typically involve self-criticism and shame.^16^ Notably, compassionate skills and attributes can be learnt and developed, increasing one’s ability to receive compassion from the self and others.^17^ In supporting a process of natural exposure and healing, SC is associated with reduced barriers to support-seeking as compassionate individuals are less likely to avoid distressing emotions.^11^ Accordingly, improved psychological outcomes, acceptability and feasibility have been reported following SC-focused interventions in PTSD, AUD and veteran populations, including use of self-administered workbooks,^19^ compassion-focused meditation^20^ and manual-guided group therapy.^11^ The wider clinical use of SC-focused digital health interventions also implies further scope for delivery,^21^ and saliently, compassion can take a strong, agentic form, providing resilience when confronting perceived threat or injustice, characterised as fierce self-compassion (FSC).^13^

Despite these benefits, the authors found no primary research exploring veterans’ SC experiences in relation to their alcohol use, and a largely quantitative body of related literature, reflecting an apparent paucity of veteran-related insight in this context.

## Methods

A qualitative approach was used to understand the lived experience of SC for UK veterans in the context of their relationships with AUD and recovery. Interpretative phenomenological analysis (IPA) emphasised the centrality of personal experience, including how things appear as interpreted from the individual perspective.^22^ Incorporating the researcher’s beliefs and values as inherent to interpretation, the development of themes, representing participants’ identities, reflected meaning not transparently available.^22^ Consistent with paradigms of addiction research which focus on ‘being’, IPA has demonstrated efficacy in exploring the lived experience of veterans.^23^

### Setting

Participants were recruited via a gatekeeper from a specialist UK addiction recovery centre, self-identifying as veterans, with lived experience of AUD (table 1) and completing the centre’s rehabilitation programme.

**Table 1:** Participant’s demographics

Participant	Branch of service	Gender	Age bracket	Years of service	Tours of duty	Longest tour duration (months)	Years since military discharge
1	Army	Male	35–45	10–20	4+	6	3–5
2	Army	Male	35–45	5–10	4+	6	10–20
3	Army	Female	35–45	20+	4+	7	>1
4	Army	Male	45–55	20+	3	12	10–20
5	Army	Male	35–45	10–20	2	6	5–10

### Sample

Inclusion criteria for the study included (1) being an adult UK veteran with at least 6 months’ military service, to facilitate rich discourse around military culture and transition, while respectfully noting the UK ‘veteran’ definition as 1-day service; (2) self-identifying with lived experience of AUD, treatment and/or recovery; (3) fluency in English; and (4) clinical stability. As an exploratory piece of research, five participants was deemed appropriate to provide sufficient information power for a study with a highly specific aim, supported by a strong interview dialogue.^24^ IPA uses characteristically small samples to enable detailed analysis and give a full appreciation to each participant’s account.^21^ Personal details were redacted or pseudonymised to maintain confidentiality.

### Data collection

A semistructured interview schedule (online supplemental appendix A) was developed with introductory questions to facilitate rapport, build trust and address potential barriers or stigma.^1^ Once established, the interviews became more conceptual in nature with questions derived from pertinent literature, exploring identity and help-seeking in the context of military culture, transition, relationships and alcohol use.^15^,^7^ Closing questions provided the opportunity to discuss topics not previously covered or considered salient to participants. Relevance, applicability and co-production were assured through discussion with veteran colleagues and the charity’s gatekeeper. Face-to-face interviews were conducted in the centre’s private room by the lead author (LJB), facilitating psychological safety and confidentiality.^22^ All participants were asked the same 10 questions, with prompts such as ‘how did that feel?’ and ‘what did that mean to you?’ eliciting further exploration if required. Each interview, lasting 45–60 min, was audio-recorded and transcribed verbatim.

### Data analysis

A detailed examination of veterans’ lived experience was undertaken using established IPA guidance.^22^ Transcripts were read and annotated to understand individual perspectives before attempting to interpret participants’ sense-making of meaningful events and people.^22^ Identified codes were observed and organised, allowing psychological conceptualisation while remaining grounded in participants’ meaning, developing an interpretive dialogue between researcher, data and theory. Adhering to the established criteria,^25^ prolonged engagement, co-production, journaling and supervision improved trustworthiness with a ‘common language’ developed to respect military norms and avoid researcher separation. Once all interviews had been coded, conceptual patterns were established across the interviews to construct themes.

## Findings

### Themes

Two superordinate themes were identified from the interviews (table 2). The first captures how participants made sense of SC and alcohol use to experience safety within their evolving lifeworlds. The second reflects the significance of finding purpose and re-engaging with military values within their recovery experiences. Quotes represent participants’ voices within this interpretation.

**Table 2:** Summary of key themes and subthemes

Superordinate themes	
Searching for safety	Healing with honour
*Subordinate themes*	*Subordinate themes*
Escaping unacceptance	Kin and kind
Safety in structure	Healing through outrage
	Compassionate flows

### Superordinate theme 1: Searching for safety

Alcohol was often experienced as an escape from ACE and low self-esteem, and later as a ‘medicine’ to ameliorate unresolved trauma and disappointment. Initially encouraged by the military, alcohol represented a ‘mask’ participants could use to hide or feel accepted.

#### Escaping unacceptance

Participants often made sense of alcohol use as an escape from isolation or not feeling ‘wanted or worthy’, inferring a paucity of SC and self-criticism which may originate from ACE.

I should have noticed growing up as a kid, the state she [Mum] used to get into, the absolute states. I used to pick her up from outside as a child, covered in mud, and cut her face, and I was in them states. I was doing exactly the same thing but telling myself ‘I’m alright.’ (Participant 3)

Experiencing parental affection as inconsistent or conditional, participants described a role reversal, reflecting an enduring disproportionate sense of responsibility during parental abdication.

I drank because, um, I wanted to fit in. It gave me confidence. It filled me with everything I needed to attack any situation […] a thousand masks for every situation […] full of confidence and just one of the lads, one of the soldiers, whatever. This is the kind of person we want. (Participant 3)

Alcohol was ubiquitously experienced as facilitating belonging needs, embodying a coveted role as ‘one of the lads’ and meeting the need for acceptance within the masculine military culture, particularly for a female soldier.

In this context, alcohol provides a ‘mask’ of acceptability and confidence, reducing feelings of isolation.

#### Safety in structure

Experienced as a family, the military offered safety through its clear structure and rules, as alcohol was experienced as acceptable, facilitating connection.

People, you know, didn’t look twice if you were pissed, as long as you turned up on time for work and that, and they’d almost cover for you as long as you were there. (Participant 4)

Subsequently, alcohol appeared caveated by implicit rules, implying conditional acceptance of the alcohol-using self.

In contrast, participants thrived in situations requiring explicit rule adherence.

You’re in charge of people, so you’re concentrating on, just living. You know, just being alive […] You wouldn’t add alcohol to the mix, or drugs, it just wouldn’t work. You wouldn’t survive. (Participant 5)

Deployment, governed by unambiguous boundaries, offered an identity where participants could embody caretaker or leadership roles, fulfilling purpose and belonging needs, negating the need for alcohol.

As rules appeared inconsistent or easily transgressed, acceptance was experienced as conditional, leading to isolation as alcohol interfered with military duties.

How they [army] get you is, you’re failing the service test. It’s not being fit for duty because you’re under the influence of alcohol. (Participant 3)

To make sense of this perceived injustice, perceptions of the military appear to fracture from a supportive brotherhood to depersonalising and reproachful.

A further way in which participants depicted the concept of safety was describing alcohol as a medicine used to ameliorate grief, trauma and isolation.

Drugs and drink was like a bandage that kept that, you know, kept the seal on it. (Participant 5)

Alcohol was also experienced as a safety strategy, enabling avoidance or distraction from intolerable affect.

I couldn’t talk to anyone. I didn’t feel understood, angry, and I thought just by using or drinking it just … sort of escapism. Just got me out of everything … got me out of me. (Participant 1)

Ultimately, alcohol provided an ‘escape’ from the self, perceived as inadequate or unwanted.

### Superordinate theme 2: Healing with honour

The second key theme reflects the significance of finding purpose and re-engaging with military values, such as integrity, courage and discipline. Although SC was considered challenging, bidirectional compassion, experienced through role modelling and trusted relationships, offered a gateway to SC.

#### Kin and kind

Participants described the significance of shared, military-conducive values.

You can’t have a brotherhood like you’ve got when you’ve got a veteran by the side of you […] that [AA] only stops at the end of a group. But where the veterans’ community we have now, it doesn’t stop. (Participant 5)

The recovery environment initially appeared to support participants’ identities as separate from civilians, perceived as having limited or conditional understanding.

They [therapists] build the trust up. It takes a hell of a lot to build the trust up. They were there for me and they didn’t promise me something they couldn’t do. They delivered. (Participant 4)

Later through identifying shared values, participants experienced connection beyond military identities, fulfilling acceptance needs with commitment from others to honour trust and integrity.

#### Healing through outrage

SC was sometimes experienced as incongruent or uncomfortable, particularly regarding morally transgressive events.

Part of me will have a smile on my face and part of me will weep. (Participant 2)

This dichotomy was encapsulated by one participant’s challenge to understand his compassion, described as ‘a moment of weakness’ leading to ‘genocide’.

I’ve always been hard on myself … but I think that’s part of my character defect … the thing is, when I was serving I’ve never lost anyone under my command and I put that down to being a bit of a perfectionist. (Participant 1)

Conversely, self-criticism was often deemed acceptable and valuable, providing safety for others.

She [daughter] might have forgiven me but I won’t forgive myself. And that drives me not to drink again. (Participant 4)

Inferring a fear of SC, self-criticism and shame appear protective, maintaining sobriety, or as an act of contrition.

Worst thing you can do to a veteran or a soldier … is promising them the world and not delivering anything, you know. If you can’t do it, don’t fucking say you can do it. (Participant 4)

Despite challenges experiencing SC, participants appeared to readily embody healthy anger in fighting for survival and against injustice.

#### Compassionate role modelling

Compassion from others appeared to denote a catalyst in recovery and a gateway to SC, providing evidence of participants’ inherent self-worth.

I never thought it was possible for me to be anything other than what I was … a soldier. But people have shown me you can be whatever you want to be. (Participant 5)

Participants appeared to experience self-worth when reflected by valued peers and observing their recovery, which inspired hope for a previously unimaginable future.

When you’ve learned the tools that you’ve learned, it’s not difficult. (Participant 5)

Adopting a ‘fake it ‘till I make it’ approach, participants could follow a familiar framework and model behaviours before experiencing positive feelings for themselves.

Once I’d taken alcohol out of the equation, I was left with me. (Participant 2)

Within a compassion-focused recovery environment, participants appeared able to experience themselves as separate to the alcohol-using self.

I’ve not been a bad person. I’ve just been fucking trapped under alcohol. (Participant 3)

In this context, participants exhibit SC in re-evaluating actions away from shame-based self-perceptions or viewing the self as inherently ‘bad’.

## Discussion

This study examined UK veterans’ lived experience of SC in the context of AUD and recovery analysed in the context of the bipolar elements of SC.

### Self-criticism and Self-kindness

Reflecting findings depicted within the theme ‘Escaping unacceptance’ and social identity theory,^26^ enlistment appears to provide favourable self-evaluation,^27^ addressing the self-criticism often associated with ACEs and maladaptive self-concepts.^13^ Accordingly, caretaker or leadership roles may provide an identity congruent with military values, addressing ineffective care-eliciting strategies following ‘parentification’, or reversal of childhood roles.^28^ Although alcohol initially offers a safe and acceptable identity^29^ to challenge self-criticism, prolonged use may inhibit self-kindness by suppressing affect.^30^ As reflected within ‘Safety in structure’, the coherent identity available during deployment provides a framework to understand self-relevant information^31^ as ‘worthy’ demands and challenges negate the need for alcohol.

Although we cannot generalise, in drinking to become ‘one of the lads’, alcohol may facilitate an acceptable masculine identity (‘mask’) for the female participant. As female veterans are more likely to experience ACEs, and at greater risk of AUD,^4^ future research should place emphasis specifically on their idiographic lived experience of serving in a traditionally masculine environment.

### Isolation and Common humanity

As paradoxically suggested within ‘Kin and kind’, fear of SC, often associated with attachment trauma,^12^ may explain participants’ desire to covet strong military-like bonds while remaining wary of civilians.^6^ Reflected within ‘Safety in structure’, transition experiences may represent a threat to identity and connection,^27^ leading to self-directed shame.^12^ Accordingly, participants’ sense-making appears to fracture between perceptions of a supportive military brotherhood and disloyal authoritative structure. As historical actions may be re-perceived as morally transgressive, cognitive biases may reflect learnt responses to loss of meaning, isolation and shame.^9^

Although group acceptance initially appears conditional, participants seem to evolve from addiction towards recovery self-concepts^30^ when supported by a secure therapeutic base.^12^ Sharing the affinity of suffering within the recovery community,^30^ reflecting a common humanity,^18^ participants appear open to support services characterised by trust, understanding and compassionate narratives.^15^ Integrated recovery groups^7^ may also address concerns regarding military re-engagement as a barrier to transition through reinforcing past identities.^32^

### Overidentification and Mindfulness

Although shame is often considered a barrier to recovery,^15^ self-criticism was experienced as protective and motivational,^13^ as depicted within ‘Healing through outrage’. Accordingly, participants’ self-critical overidentification, often related to morally transgressive events, may suggest a functional view of shame in response to threatened social belonging, and a driver to repair self-image.^12^

In ‘Compassionate flows’, compassion was experienced as relational and appeared to activate participants’ soothing system in response to perceived threat, consistent with social mentality theory.^17^ In flowing bidirectionally between the self and others, relational compassion appears to support SC and meaning-making in reducing perceived differences. As experiences were accepted and normalised through modelling vulnerability, participants were able to build connection and feel worthy. A compassionate peer-led recovery approach appears to support a mindful, balanced view of actions which reframes shame from ‘being bad’ to ‘doing bad things’.^15^

Although SC may be considered weak or gentle, and therefore incongruent with self-concept, as suggested within ‘Healing through outrage’, participants embodied the healthy anger and self-protection characteristic of FSC.^13^ Facilitating the transition from shame and self-criticism into righteous outrage, pertinent in trauma contexts, FSC may help reframe support-seeking as congruent with masculine military norms. Furthermore, as social identity may fluctuate depending on self and group evaluations,^30^ FSC may help veterans realise their strength and value in identifying what defines them outside of the collective military identity.^27^

As participants sought to escape self-criticism and find purpose and identity, a compassionate ‘fake it till I make it’ and/or peer-led recovery approach appears to support a balanced self-evaluation, congruent with military values. In providing an acceptable form of exposure therapy^11^ and improving symptom recognition associated with help-seeking, compassion-based interventions may support service leavers to normalise the changes experienced during transition, which appear to ease over time.^5^ Drawing on the combat veteran paradox,^7^ in suggesting all veterans may benefit from interventions to understand their new worldview, military leadership involvement may also address cultural stigma and poor engagement. As SC may feel incongruent, interventions reframed as FSC may support veterans to access moral outrage and healthy anger to fight healthcare issues ‘like a solider’, reframing support-seeking as congruent with military norms, helping veterans realise their strength, value and individuality.

While initially wary, veterans demonstrated a readiness to experience trusted therapeutic relationships with healthcare providers who made the effort to understand their experience. Accordingly, an e-learning module may build on NHS pledges to improve veterans’ healthcare experiences and provider knowledge, as a feasible and acceptable tool.^33^

## Conclusion

This study provides a unique, in-depth perspective of UK veterans’ experience of SC within the context of AUD and recovery, with IPA ensuring veterans’ voices were central. This contributes to an improved understanding of veterans’ healthcare needs and experience, as advocated by government and NHS policy, and benefits from a female perspective as notably under-represented. Findings support wider literature purporting the role of trauma and identity in veterans’ support-seeking and affect regulation, reflecting the symbiotic relationship between shame and AUD which impacts veterans’ experience of SC. Acknowledging affect regulation strategies as feasible within veteran and AUD populations, SC may be peer-modelled or reframed as FSC to improve acceptability. Clinicians may also benefit from understanding veterans’ unique needs and experiences to improve integration and transition.

While not aiming to generalise, these qualitative findings may be transferable to populations characterised by the sample criteria and support existing theories including the combat veteran paradox, while a larger study may enable further analysis and focus on the experience of UK female veterans to address geographic and gender-based disparities in military research.

### Reflexivity

The first author (LJB) is a civilian nurse specialising in alcohol-related care, experienced in supporting military veterans with complex mental health needs, aspiring to improve veteran healthcare pathways and experiences. AB is a senior lecturer specialising in health and well-being research. JK is a senior lecturer specialising in qualitative methods with experience of researching long-term recovery from substance misuse. As building trust was essential in understanding participants’ experiences, while ‘bracketing’ the nursing role which seeks to alleviate distress, a degree of vulnerability was proffered to address any power asymmetry or ‘outsider’ status. Reflexive journaling and supervision were used to interpret the experience as co-constructed, with participants empowered in selecting what to reveal.

## supplementary material

10.1136/military-2023-002383 online supplemental file 1

## Data Availability

Data are available upon reasonable request.
