# Supplementary material for: Courage, camaraderie and compassion: a qualitative exploration into UK military veterans’ experiences of self-compassion within the context of alcohol use disorders and recovery
Source: BMJ Mil Health. 2023 Jul 25;171(1):e002383. doi: 10.1136/military-2023-002383 (PMC11877041; doi:10.1136/military-2023-002383)
Supplement: online supplemental file 1 [file military-171-1-s001.pdf]

Appendix A

Example Interview Schedule

1. Can you tell me about your role within the military (descriptive/low risk/warm up)

(Prompt: how did you view yourself? leader, comrade, brother, warrior? How did it make you feel?)

2. How does that compare to your life as a civilian? How do you feel about this?

(Prompt: acceptance? Identity? Impact upon life?)

3. Have you approached a support service regarding alcohol?

(Prompt: what prompted you? Or what prevented you?)

4. How would you describe your relationship with alcohol (descriptive/introducing alcohol)? (Kiernan et al., 2013; Battles et al., 2019)

(Prompt: Controlled? improved? Dependent? Frustrated? Growth?)

5. How has your relationship with alcohol changed throughout the different stages of your life? (Kiernan et al., 2013; Koch, 2019)

(Prompt: pre-enlistment? Transition? Reasons for drinking? Drinking pattern?)

6. Can you tell me about the role alcohol has played in your life? What does that feel like? (Koch, 2019; Guerrero et al., 2021)

(Prompt: Medicinal? Coping? Painful? Relapse? Triggers? What made those experiences positive/negative? How did you feel?)

7. Can you tell me about a significant or meaningful experience you've encountered during alcohol treatment or your experience of recovery? (Koch, 2019; Eaton et al., 2020; Guerrero et al., 2021)

(Prompt: practitioner? Environment? breakthrough? Painful? Relapse? Triggers? What made those experiences positive/negative? How did you feel?)

8. What about more/less positive experiences you've had? How did that feel? (Forkus et al., 2019)

(Prompt: What do you think made those experiences positive/negative?)

9. What impact do you think these experiences have had upon your life and your feelings about yourself and others? (Battles et al., 2019; Ahern et al., 2015)

(Prompt: relationships? Identity, strength, challenge? Isolation? Growth?)

10. What do you think you have learned from your journey with alcohol that could help a friend in similar situation? What does that feel like?

(prompt: sense of self? useful? Part of something)
